# The Increased lncRNA MIR503HG in Preeclampsia Modulated Trophoblast Cell Proliferation, Invasion, and Migration via Regulating Matrix Metalloproteinases and NF-*κ*B Signaling

**DOI:** 10.1155/2019/4976845

**Published:** 2019-07-30

**Authors:** Dan Cheng, Shan Jiang, Jiao Chen, Jie Li, Liangfei Ao, Ying Zhang

**Affiliations:** ^1^Center for Reproductive Medicine, Renmin Hospital of Wuhan University, Hubei Clinic Research Center for Assisted Reproductive Technology and Embryonic Development, Wuhan, 430060 Hebei Province, China; ^2^Department of Dermatology, Renmin Hospital of Wuhan University, Wuhan, 430060 Hebei Province, China

## Abstract

**Background:**

Preeclampsia (PE) is a pregnancy-related syndrome characterized by hypertension and proteinuria after the 20^th^ week of gestation. The long noncoding RNAs (lncRNAs) have been recently discovered for their roles in the pathogenesis of PE. This study is aimed at determining the expression of lncRNA MIR503 host gene (MIR503HG) in PE placental tissues and exploring the molecular mechanism underlying MIR503HG-mediated trophoblast cell proliferation, invasion, and migration.

**Methods:**

The expression level of MIR503HG in placental tissues, HTR-8/SVneo, and JEG3 cells was determined by quantitative real-time PCR; western blot detected the relevant protein expression levels in HTR-8/SVneo and JEG3 cells; flow cytometry determined cell apoptosis and cell cycle of HTR-8/SVneo and JEG3 cells; trophoblast cell proliferation, invasion, and migration of HTR-8/SVneo and JEG3 cells were measured by CCK-8, transwell invasion, and wound healing assays, respectively.

**Results:**

The highly expressed MIR503HG was detected in PE placental tissues compared to normal placental tissues. MIR503HG overexpression suppressed cell proliferation, invasion, and migration of HTR-8/SVneo and JEG3 cells, while knockdown of MIR503HG increased trophoblast cell proliferation, invasion, and migration. Flow cytometry results showed that MIR503HG overexpression induced apoptosis and caused cell cycle arrest at the G_0_/G_1_ phase, while MIR503HG knockdown had the opposite actions in HTR-8/SVneo and JEG3 cells. Western blot assay results showed that MIR503HG overexpression suppressed the matrix metalloproteinase-2/-9 and the snail protein expression and increased the E-cadherin expression in trophoblast cells. In addition, MIR503HG overexpression suppressed the NF-*κ*B signaling pathway by inhibiting the phosphorylation of I*κ*B*α* and the nuclear translocation of NF-*κ*B signaling subunit p65. On the other hand, MIR503HG knockdown played an opposite role in these protein expression levels.

**Conclusion:**

Our results showed that MIR503HG inhibited the proliferation, invasion, and migration of HTR-8/SVneo and JEG3 cells, which may be related to the pathogenesis of PE.

## 1. Introduction

Preeclampsia (PE) is a pregnancy-related syndrome characterized by hypertension and proteinuria after the 20^th^ week of gestation [1], and PE affects about 4% of pregnancies and accounts for more than 15% maternal mortality worldwide [2]. Normal proliferation and differentiation of human placental trophoblasts are important in maintaining the proper function of the placenta [3]. Inadequate trophoblast cell invasion and migration was found to be associated with PE [4]. The deficiencies in trophoblast invasion and migration are the main contributors of the PE pathogenesis, and in the early stage of PE, the inadequate invasion of trophoblast cells leads to the impairment of vascular remodeling and the decrease in the circulation of the early placenta, which consequently causes placental ischemia after the 20^th^ week of gestation [5]. In addition, increased apoptosis of placental trophoblasts within the uterine wall was also demonstrated to involve in the pathogenesis of PE [6]. Up to date, proangiogenic and antiangiogenic factors in the circulation, including placental growth factor, endoglin, and fms-related tyrosine kinase 1, were indicated to play important roles in the pathogenesis of PE [7]. However, the molecular mechanisms underlying PE pathogenesis remain unclear.

Long noncoding RNAs (lncRNAs) are RNA species more than 200 nucleotides in length and lack protein-coding potential [8]. lncRNAs have been found to play important roles in the pathogenesis of various diseases, particularly in cancer [9]. Recently, multiple studies showed the differential expression of lncRNAs in the PE placentas, suggesting that lncRNA may contribute to the pathogenesis of PE [10]. The loss of the lncRNA H19 gene imprinting in the placental tissues of PE patients was found to be associated with severe hypertension, which may contribute to the pathogenic process of PE [11]. The lncRNA-maternally expressed gene 3 (MEG3) was found to be downregulated in placental samples from PE patients, and abnormal levels of MEG3 were shown to result in cellular dysfunctions of HTR-8/SVneo and JEG3 trophoblast cells [12]. Zou et al. identified the upregulation of lncRNA SPRY4 intronic transcript 1 (SPRY4-IT1) in PE placental tissues, and SPRY4-IT1 regulated trophoblast cell invasion and migration by affecting the epithelial-mesenchymal transition [13]. Xu et al. also found that lncRNA taurine upregulated 1 modulated proliferation in trophoblast cells via epigenetic suppression of the Rho family GTPase 3 [14]. Recent studies showed that placenta-enriched lncRNA MIR503 host gene (MIR503HG) decreased the migration and invasion potential of JEG3 cell line [15], suggesting a potential role for MIR503HG in the PE. However, the exact role of MIR503HG in the pathogenesis of PE has not been investigated so far.

In the present study, we intended to discover the possible roles of MIR503HG in trophoblast proliferation, invasion, and migration. The elevation of placental MIR503HG was identified in severe PE placental tissues. In addition, the effects of MIR503HG on cellular functions were also explored using *in vitro* studies.

## 2. Materials and Methods

### 2.1. Sample Collection

Forty severe PE pregnant subjects and 40 normal pregnant subjects were recruited at the moment of admission to Renmin Hospital of Wuhan University. Diagnosis of severe PE was based on the definition in Williams Obstetrics (23^rd^ edition) [16]. Pregnant patients (more than 20 weeks of gestation) with no history of preexisting/chronic hypertension exhibited systolic/diastolic blood pressure ≥ 160/110 mmHg on 2 separate readings, proteinuria measurement of 1+ or more times, or 24 h urine protein collection with ≥300 mg. Subjects with disorders such as diabetes, lupus, urinary tract infection, or chronic renal disease were excluded from this study. All pregnancies were treated by elective cesarean delivery in the absence of labor, and the placental tissues were collected within 1 h of cesarean birth and stored in -80°C for further use. All the research procedures were approved by the Ethics Committee of Renmin Hospital of Wuhan University, and informed consent was obtained from all the participated subjects.

### 2.2. Cell Line and Cell Culture

The human trophoblast cell lines including HTR-8/SVneo and JEG3 cells (HTR-8/SVneo: derived by transfecting the cells that grew out of the chorionic villi explants of human first-trimester placenta with the gene encoding for simian virus 40 large T antigen; JEG3 cells: derived from a human choriocarcinoma and presented many of the biological and biochemical characteristics associated with syncytiotrophoblasts) were purchased from the American Type Culture Collection company (Manassas, VA, USA) and cultured in RPMI 1640 medium (Thermo Fisher Scientific, Waltham, CA, USA) containing 10% fetal bovine serum (FBS; Thermo Fisher Scientific), 100 *μ*g/ml streptomycin, and 100 UI/ml penicillin. Cells were incubated at 37°C with 5% CO_2_ and routinely passaged every 3 d.

### 2.3. Plasmids, siRNAs, and Cell Transfection

The pcDNA3.1 vector was used to generate MIR503HG-overexpressing vector, and the empty vector, pcDNA3.1, and MIR503HG-overexpressing vector (pcDNA3.1-MIR503HG) were purchased from GeneChem (Shanghai, China). The siRNAs targeting MIR503HG (si-MIR503HG) and the scrambled negative controls (si-NC) were purchased from RiboBio (Guangzhou, China). For plasmid and siRNA transfection, trophoblast cells of a confluent cell monolayer were transiently transfected with plasmids (20 *μ*g) or siRNAs (100 nM) using Lipofectamine 2000 (Invitrogen, Carlsbad, CA, USA) according to the manufacturer's instruction. At 24 h after transfection, cells were processed for further experimentation.

### 2.4. Quantitative Real-Time PCR (qRT-PCR)

Total RNA from placental tissues and cells was extracted using TRIzol reagent (Invitrogen), and the synthesis of cDNA was performed by the reverse transcription kit (Takara, Dalian, China). The amplification of cDNA was performed using Power SYBR green (Takara) in a reaction mix for real-time PCR on the ABI7900 system (Applied Biosystems, Waltham, MA, USA). The gene expression levels were quantified by the comparative Ct method, and analytical data were normalized to the mRNA expression level of glyceraldehyde-3-phosphate dehydrogenase. The primers for the real-time PCR were as follows: MIR503HG, forward, 5′-CTTGAAGGCATCCAGCATCTC-3′ and reverse, 5′-TTGGGACACTTGGGTGGTTTT-3′; GAPDH, forward, 5′-CGCTCTCTGCTCCTCCTGTTC-3′ and reverse, 5′-ATCCGTTGACTCCGACCTTCAC-3′.

### 2.5. Cell Counting Kit-8 (CCK-8) Assay

The transfected cells were seeded in the 96-well plates, and CCK-8 assays were performed at 0, 24, 48, 72, and 96 h after seeding. For the assay, culture medium was replaced with 100 *μ*l of CCK-8 solution (Beyotime, Beijing, China) and incubated for 2 h at room temperature. The cell proliferation index was detected by measuring optical density (OD) values at the wavelength of 450 nm via the Microplate Reader (Thermo Fisher Scientific).

### 2.6. Transwell Invasion Assay

The transfected cells were suspended in 200 *μ*l RPMI 1640 medium without FBS and seeded in the upper compartment Transwell inserts (8 *μ*m in pore size, Millipore, Burlington, USA) coated with Matrigel, whereas the lower chamber was filled with full medium. After 24 h incubation at 37°C, noninvasive cells attaching to the top side of the inserts were cleaned by cotton swabs and the invasive cells were fixed with 4% paraformaldehyde and stained with 0.5% crystal violet for 10 min at room temperature. Stained cells were photographed and counted by using a light microscope (Leica, Heidelberg, Germany).

### 2.7. Wound Healing Assay

The transfected cells were seeded in the 6-well plates and subjected to serum starvation for 4 h. Then, a straight scratch in a cell monolayer was made by a sterile 200 *μ*l pipette tip to simulate a wound. The scratched monolayer was then rinsed twice with a serum-free medium gently and allowed to heal in a complete medium for 24 h. The wound width was photographed using an inverted microscope at 0 and 24 h after wound formation, respectively. Cell migration was evaluated by the measurement of % wound closure.

### 2.8. Flow Cytometry

For cell cycle determination, transfected cells were collected and washed with cold phosphate-buffered saline (PBS), and cells were fixed with 70% ethanol. Thereafter, cells were stained with propidium iodide (PI) staining solution in PBS for 15 min at 37°C. Stained cells were analyzed for cell cycle distribution by using BD FACSCanto II (BD Biosciences, San Jose, CA, USA).

For cell apoptosis determination, apoptotic cells were detected by Annexin V-FITC/PI double staining using the Annexin V-FITC Apoptosis Detection Kit (Thermo Fisher Scientific) according to the manufacturer's instruction. Briefly, transfected cells were collected and washed with cold PBS, and cells then were stained with Annexin V-FITC and PI in binding buffer for 10 min. The stained cells were then analyzed using BD FACSCanto (BD Biosciences).

### 2.9. Western Blot

Proteins from cells were extracted using radioimmunoprecipitation assay lysis buffer (Beyotime), and the proteins in nuclear fractions were prepared with the NE-PER extraction kit (Thermo Fisher Scientific) according to the manufacturer's instruction. The extracted protein was resolved in sodium dodecyl sulfate-polyacrylamide gel electrophoresis and transferred to polyvinylidene fluoride (PVDF) membranes. The PVDF membranes were blocked with 5% skimmed milk and incubated with appropriated primary antibodies at 4°C overnight. Thereafter, the PVDF membranes were incubated with horseradish peroxidase-conjugated secondary antibodies. Protein blots were visualized by using the ECL-Plus western blotting detection system (Thermo Fisher Scientific). The used primary antibodies include rabbit polyclonal antibodies for matrix metalloproteinase-2 (MMP2; 1 : 1500; Cell Signaling Technology, Danvers, MA, USA), MMP9 (1 : 1500; Cell Signaling Technology), snail (1 : 1000; Abcam, Cambridge, UK), E-cadherin (1 : 1000; Abcam), p65 (1 : 500; Cell Signaling Technology), I*κ*B*α* (1 : 500; Cell Signaling Technology), and lamin A (1 : 1500; Abcam) and rabbit monoclonal antibodies for phosphorylated I*κ*B*α* (1 : 1000; Cell Signaling Technology) and *β*-actin (1 : 2000; Abcam).

### 2.10. Statistical Analysis

Statistical analysis was performed using SPSS 19.0 statistical software (IBM SPSS, Armonk, NY, USA). All experiments were performed with 3 technical repeats, deriving from at least 3 individual experiments, and the data were presented as mean ± standard deviation. Student's *t*-test was performed to estimate the significance of the differences caused by treatments relative to their corresponding controls. *P* < 0.05 was considered statistically significant.

## 3. Results

### 3.1. The Expression of MIR503HG Was Upregulated in PE Placental Specimens

**Figure 1:**
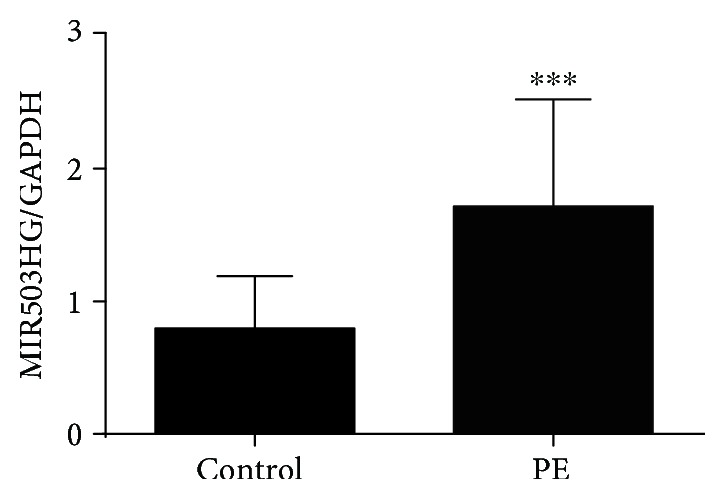
The expression level of MIR503HG in placental tissues. The relative expression level of MIR503HG in placental tissues from normal pregnant women (*n* = 40) and women with severe PE (*n* = 40) was measured by qRT-PCR assay. ^∗∗∗^*P* < 0.001 compared to the control group.

The clinical characteristics of the pregnant women who participated in this study are shown in Table 1. The systolic blood pressure and diastolic blood pressure were significantly higher in the PE group in comparison with the control group. Furthermore, proteinuria was detected in the PE group but not in the control group. In addition, the birth weight of the newborns in the PE group was lower than that in the control group. The expression of MIR503HG in the placental tissues was determined by qRT-PCR assay, and the expression of MIR503HG in the placental tissues from the PE group was significantly higher than that from the control group (Figure 1), suggesting the potential roles of MIR503HG in PE.

### 3.2. MIR503HG Suppressed Trophoblast Cell Proliferation, Invasion, and Migration

**Figure 2:**
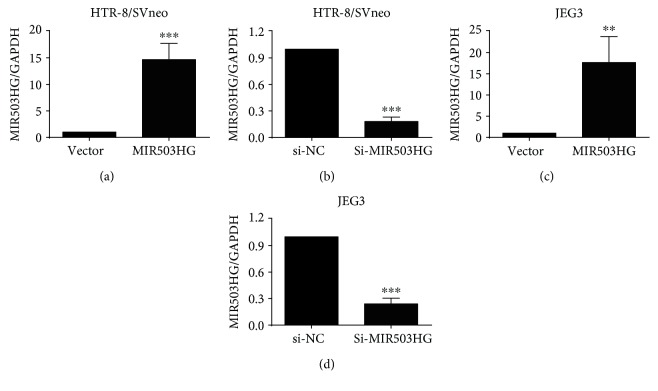
The expression of MIR503HG in trophoblast cells transfected with plasmids or siRNA. (a, b) qRT-PCR assays were implemented to measure the expression level of MIR503HG in HTR-8/SVneo cells transfected with pcDNA 3.1 (vector) or pcDNA3.1-MIR503HG (MIR503HG) and scrambled siRNA (si-NC) or MIR503HG siRNA (si-MIR503HG). (c, d) qRT-PCR assays were also performed in JEG3 cells after transfecting with the above-mentioned plasmids or siRNAs. *N* = 3, ^∗∗^*P* < 0.01 and ^∗∗∗^*P* < 0.001 compared to the corresponding control groups.

The overexpression of MIR503HG in HTR-8/SVneo and JEG3 cells was carried out by transfecting trophoblast cells with pcDNA3.1-MIR503HG, and the expression levels of MIR503HG in pcDNA3.1-MIR503HG-transfected trophoblast cells were significantly higher than that in pcDNA3.1 vector-transfected trophoblast cells (Figures 2(a) and 2(c)). The knockdown of MIR503HG in HTR-8/SVneo and JEG3 cells was carried out by transfecting trophoblast cells with MIR503HG siRNA (si-MIR503HG), and the expression levels of MIR503HG in si-MIR503HG-transfected trophoblast cells were significantly lower than that in si-NC-transfected cells (Figures 2(b) and 2(d)).

**Figure 3:**
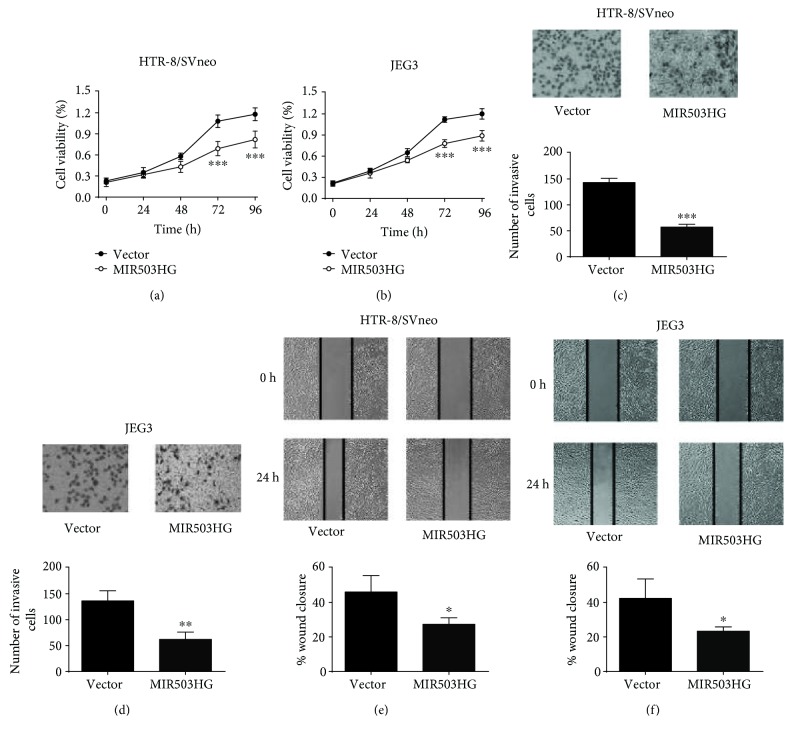
Upregulation of MIR503HG suppressed trophoblast cell proliferation, invasion, and migration. (a, b) CCK-8 assays were performed to measure the cell proliferation of HTR-8/SVneo and JEG3 cells transfected with pcDNA3.1 or pcDNA3.1-MIR503HG. (c, d) Transwell invasion assays were performed to determine the cell invasion of HTR-8/SVneo and JEG3 cells transfected with pcDNA 3.1 or pcDNA3.1-MIR503HG. (e, f) Wound healing assays were performed to determine the cell migration of HTR-8/SVneo and JEG3 cells transfected with pcDNA3.1 or pcDNA3.1-MIR503HG. *N* = 3, ^∗^*P* < 0.05, ^∗∗^*P* < 0.01, and ^∗∗∗^*P* < 0.001.

We subsequently assess the effects of MIR503HG overexpression on trophoblast cell proliferation by CCK-8 assay, and the cell proliferation of HTR-8/SVneo and JEG3 cells was significantly suppressed after pcDNA3.1-MIR503HG transfection (Figures 3(a) and 3(b)). The trophoblast invasion was assessed by Transwell invasion assay, and the number of invasive HTR-8/SVneo and JEG3 cells was significantly reduced after pcDNA3.1-MIR503HG transfection (Figures 3(c) and 3(d)). Furthermore, the cell migration was then assessed by wound healing assay, and the wound closure was significantly suppressed in HTR-8/SVneo and JEG3 cells transfected with pcDNA3.1-MIR503HG (Figures 3(e) and 3(f)).

**Figure 4:**
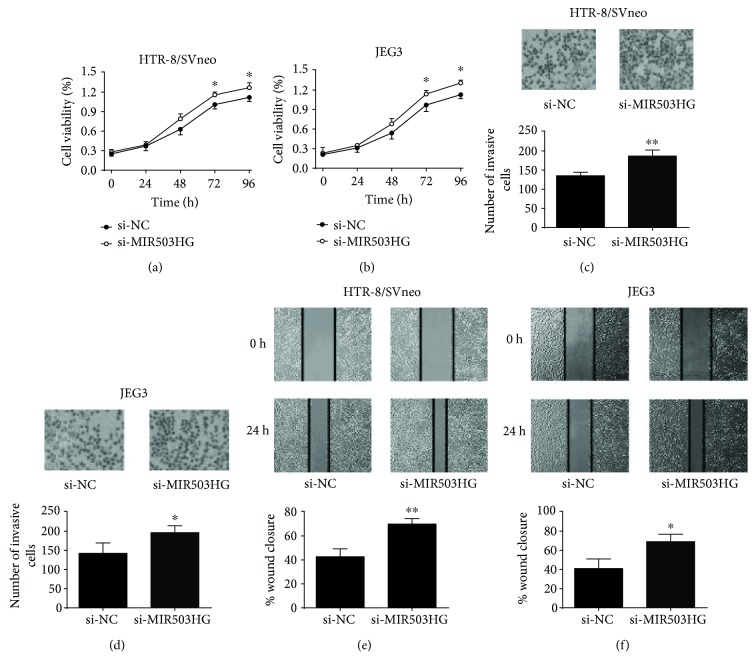
Knockdown of MIR503HG promoted trophoblast cell proliferation, invasion, and migration. (a, b) CCK-8 assays were performed to measure the cell proliferation of HTR-8/SVneo and JEG3 cells transfected with scrambled siRNA or MIR503HG siRNA. (c, d) Transwell invasion assays were performed to determine the cell invasion of HTR-8/SVneo and JEG3 cells transfected with scrambled siRNA or MIR503HG siRNA. (e, f) Wound healing assays were performed to determine the cell migration of HTR-8/SVneo and JEG3 cells transfected with scrambled siRNA or MIR503HG siRNA. *N* = 3, ^∗^*P* < 0.05 and ^∗∗^*P* < 0.01.

On the other hand, the cell proliferation, cell invasion, and cell migration of HTR-8/SVneo and JEG3 cells were significantly enhanced after MIR503HG siRNA transfection (Figures 4(a)–4(f)). Taken together, these results suggested that MIR503HG had suppressive effects on trophoblast cell proliferation, invasion, and migration.

### 3.3. The Effects of MIR503HG Overexpression on Trophoblast Cell Apoptosis and Cell Cycle

**Figure 5:**
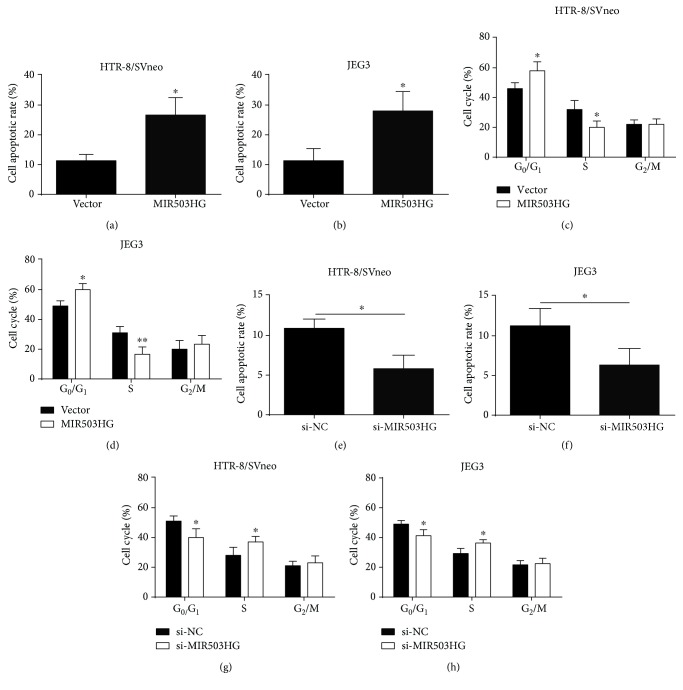
The effects of MIR503HG overexpression on trophoblast cell apoptosis and cell cycle. (a, b) Flow cytometry was performed to measure the cell apoptotic rate in HTR-8/SVneo and JEG3 cells after transfected with pcDNA 3.1 or pcDNA3.1-MIR503HG. (c, d) Flow cytometry was performed to determine the cell cycle in HTR-8/SVneo and JEG3 cells after transfected with pcDNA 3.1 or pcDNA3.1-MIR503HG. (e, f) Flow cytometry was performed to measure the cell apoptotic rate in HTR-8/SVneo and JEG3 cells after transfected with scrambled siRNA or MIR503HG siRNA. (g, h) Flow cytometry was performed to measure the cell cycle in HTR-8/SVneo and JEG3 cells after transfected with scrambled siRNA or MIR503HG siRNA. *N* = 3, ^∗^*P* < 0.05.

To explore the role of MIR503HG in trophoblast cell apoptosis and cell cycle, we performed flow cytometry experiments. The cell apoptotic rate was significantly increased in HTR-8/SVneo and JEG3 cells after transfection with pcDNA3.1-MIR503HG (Figures 5(a) and 5(b)). The cell cycle analysis showed that overexpression of MIR503HG increased cell population at the G_0_/G_1_ phase and decreased cell population at the S phase in both HTR-8/SVneo and JEG3 cells (Figures 5(c) and 5(d)). On the other hand, knockdown of MIR5053HG reduced cell apoptosis and promoted cell cycle progression in both HTR-8/SVneo and JEG3 cells (Figures 5(e)–5(h)). Collectively, the results suggested that MIR503HG induced cell apoptosis and caused cell cycle arrest at the G_0_/G_1_ phase of trophoblast cells.

### 3.4. MIR503HG Suppressed Matrix Metalloproteinases (MMPs) and Snail Protein Expression and Increased E-Cadherin Expression

**Figure 6:**
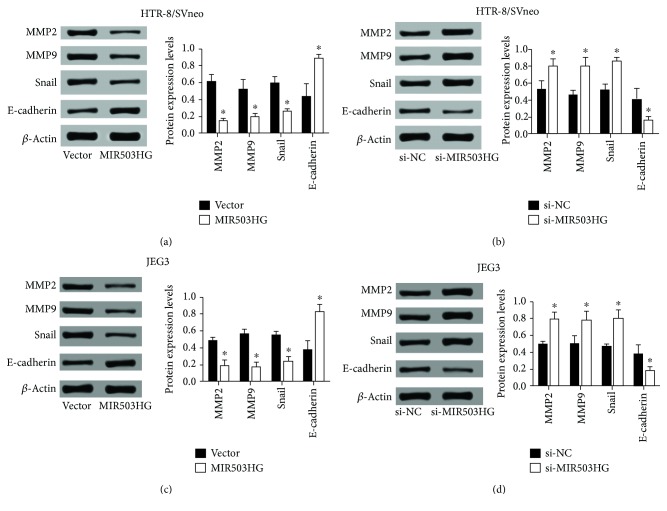
MIR503HG suppressed MMPs and snail protein expression and increased E-cadherin expression. (a, b) Western blot assays were performed to determine the protein expression levels of MMP2, MMP9, snail, and E-cadherin in HTR-8/SVneo cells transfected with pcDNA 3.1 or pcDNA3.1-MIR503HG and scrambled siRNA or MIR503HG siRNA. (c, d) Western blot assays were also performed in JEG3 cells transfected with the above-mentioned plasmids or siRNAs. *N* = 3, ^∗^*P* < 0.05.

MMPs are important mediators of vascular and uterine remodeling, and decreased expression of MMP2 and MMP9 have been suggested to involve in the hypertensive pregnancy and PE [17]. In addition, placental extravillous cytotrophoblast invasion involves a cellular transition from an epithelial to mesenchymal phenotype, and some of the epithelial-mesenchymal transition (EMT) regulators have been found to play an important role in the development of PE [18]. To further investigate the molecular mechanisms underlying MIR503HG-mediated trophoblast invasion and migration, we determined the protein levels of MMP2, MMP9, snail, and E-cadherin in trophoblast cells after MIR503HG overexpression or knockdown. As shown in Figure 6(a), overexpression of MIR503HG decreased the protein levels of MMP2, MMP9, and snail but increased the protein level of E-cadherin (Figure 6(a)), and knockdown of MIR503HG increased the protein levels of MMP2, MMP9, and snail but decreased the protein level of E-cadherin (Figure 6(b)). Consistently, similar findings regarding MMP2, MMP9, snail, and E-cadherin protein levels after MIR503HG overexpression or knockdown were demonstrated in JEG3 cells (Figures 6(c) and 6(d)).

### 3.5. MIR503HG Inhibited the NF-*κ*B Signaling Pathway

**Figure 7:**
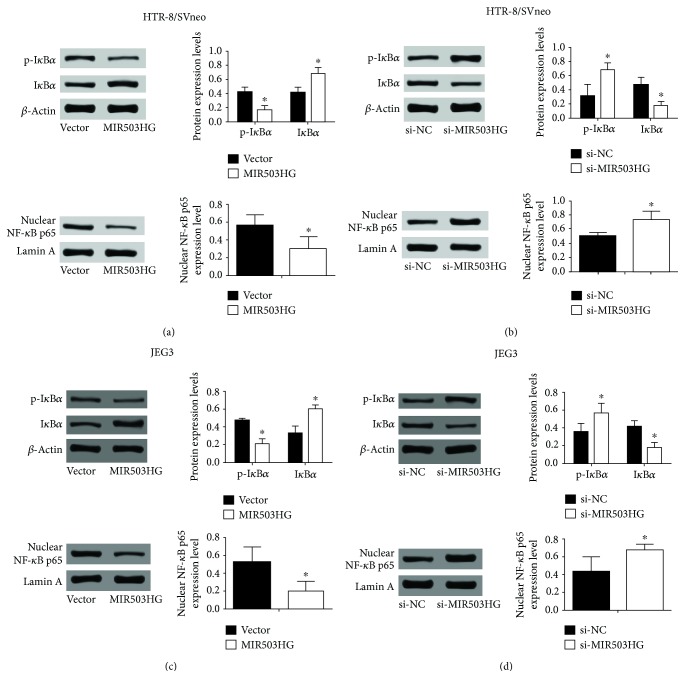
MIR503HG inhibited the NF-*κ*B signaling pathway. (a, b) Western blot assays were performed to determine the protein expression levels of p-I*κ*B*α*, I*κ*B*α*, and nuclear NF-*κ*B p65 in HTR-8/SVneo cells transfected with pcDNA 3.1 or pcDNA3.1-MIR503HG and scrambled siRNA or MIR503HG siRNA. (c, d) Western blot assays were also performed in JEG3 cells transfected with the above-mentioned plasmids or siRNAs. *N* = 3, ^∗^*P* < 0.05.

Since the NF-*κ*B signaling pathway was shown to be associated with the migratory ability of trophoblast cells, we further explored the effects of MIR503HG on NF-*κ*B signaling by using western blot to detect the I*κ*B*α* phosphorylation and nuclear NF-*κ*B p65 translocation. As shown in Figure 7(a), overexpression of MIR503HG significantly reduced I*κ*B*α* phosphorylation and inhibited nuclear NF-*κ*B p65 translocation in HTR-8/SVneo cells (Figure 7(a)), and knockdown of MIR503HG significantly increased I*κ*B*α* phosphorylation and nuclear NF-*κ*B p65 translocation in HTR-8/SVneo cells (Figure 7(b)). Consistent results regarding the effects of MIR503HG on NF-*κ*B signaling were also demonstrated in JEG3 cells (Figures 7(c) and 7(d)).

## 4. Discussion

The placenta, a transient organ forming during pregnancy, supports the fetus growth and development [19]. Abnormal placental development, particularly the inadequate invasion of trophoblast cells into the uterus and the subsequent impairment of maternal spiral artery remodeling, plays important roles in the pathogenesis of PE [20]. Recently, the placenta-enriched lncRNA MIR503HG was expressed in placental tissues, and MIR503HG decreases the migration of JEG3 cells, indicating the involvement of MIR503HG in the pathogenesis of PE [15]; in addition, the dysregulation of MIR503HG may be related to epigenetic changes due to the presence of CpG islands in the promoter regions of MIR503HG [15]. In the present study, we consistently identified the highly expressed MIR503HG in placental tissues and *in vitro* functional assays showed that MIR503HG suppressed trophoblast cell proliferation, invasion, and migration, also induced apoptosis and cell cycle arrest at the G_0_/G_1_ phase. Further mechanistic study by using western blot assay showed that MIR503HG downregulated MMPs and inhibited the NF-*κ*B signaling pathway in trophoblast cells.

Trophoblast cells physiologically invade into the uterus during pregnancy, and the trophoblast cell invasion was similar to the invasive behavior of tumor cells. Indeed, the expression level of MIR503HG was upregulated in hepatocellular carcinoma (HCC) tissues, and enhanced expression of MIR503HG inhibited HCC cell invasion and migration [21]. In the trophoblast cell line, JEG3, previous study showed that MIR503HG overexpression decreased JEG3 cell migration and invasion potential [15]. In addition, the effects of various lncRNAs on the trophoblast cell invasion and migration have been elucidated. The lncRNA plasmacytoma variant translocation 1 knockdown significantly suppressed cell proliferation, invasion, and migration and stimulated cell cycle accumulation and apoptosis [22]. The silencing of lncRNA microvascular invasion in hepatocellular carcinoma inhibited cell proliferation, invasion, migration, and angiogenesis in various trophoblast cell lines [23]. Inhibition of endogenous lncRNA activated by TGF-*β* suppressed HTR-8/SVneo cell proliferation, migration, and tube formation [24]. The trophoblast cell lines including HTR-8/SVneo and JEG3 are widely used as an *in vitro* model for studies on trophoblast cell behaviors. In the present study, we demonstrated that MIR503HG suppressed the invasion and migration in both HTR-8/SVneo and JEG3 cells, and these findings suggest that MIR503HG may contribute to the invasive and migratory capability of placental trophoblasts.

MMPs are zinc-dependent endopeptidases and play important roles in regulating cellular processes including the invasive behaviors of various types of cells [25]. MMP2 and MMP9 are important in human trophoblast cell invasion [26]. Peng et al. showed that gonadotrophin-releasing hormone regulated trophoblast invasion via Runt-related transcription factor 2-mediated MMP2 and MMP9 expression [27]. The expression levels of MMP2 and MMP9 in PE placentas were significantly downregulated when compared to normal placentas, suggesting the association between MMP2/9 and the pathogenesis of PE. In the present study, the MMP2 and MMP9 expressions were downregulated upon MIR503HG overexpression, while being upregulated upon MIR503HG knockdown in HTR-8/SVneo and JEG2 cells. Taken together, our results suggest that MMP2/9 may involve in the MIR503HG-mediated trophoblast cell invasion.

EMT is an important process in regulating the migratory behavior of trophoblast cells, and studies showed that EMT was suppressed in PE placentas [28]. In the present study, we examined the effects of MIR503HG on the protein expression of EMT-related factors including E-cadherin and snail. During the first trimester of pregnancy, E-cadherin is downregulated, which leads to the increased migratory ability of trophoblast [29]. E-cadherin can function as a negative regulator of migratory behavior of human trophoblast cells and is downregulated by cyclosporine A [30]. In the present study, we showed that MIR503HG increased the protein expression level of E-cadherin and decreased the protein expression level of snail. Collectively, these results suggest that MIR503 may contribute to EMT-related trophoblast migration.

Studies have shown that MIR503HG inhibits HCC cell invasion and migration via suppressing the NF-*κ*B signaling pathway [21], suggesting the involvement of the NF-*κ*B signaling pathway in MIR-503HG-mediated HCC cell invasion and migration. MIR503HG exerted its suppressive effects on the NF-*κ*B signaling via promoting heterogeneous nuclear ribonucleoprotein A2/B1 degradation via the ubiquitin-proteasome pathway [21]. Studies from Yu et al. showed that knockdown of notch-1 inhibited migration and invasion and suppressed the NF-*κ*B signaling pathway in trophoblast cells [31], implying the involvement of NF-*κ*B signaling in trophoblast invasion and migration. In addition, the NF-*κ*B signaling pathway was also found to regulate the MMP expression and EMT processes [32]. The activity of NF-*κ*B signaling (evaluated by immunohistochemistry) was also found to be elevated by oxidative stress in the PE women [33], while in our study, MIR503HG exerted suppressive effects on the NF-*κ*B in the trophoblast cells, and this contradiction may be attributed to differences in the cell types between different studies, which still requires further investigation. In our results, MIR503HG inhibited the phosphorylation of I*κ*B*α* and the nuclear translocation of NF-*κ*B signaling subunit p65, suggesting that NF-*κ*B signaling may involve in the inhibitory effects of MIR503HG on trophoblast cell invasion and migration.

Several limitations should be considered for the present study. Firstly, the expression of MIR503HG in the placental tissues was only determined by qRT-PCR assay, and further immunohistochemistry may be performed to confirm the expression of MIR503HG in the placental tissues. Secondly, the expression levels of MMPs were not determined in the placental tissues due to the limited placental tissues, and further studies may be performed to determine the expression of these MMPs and their correlation with the MIR503HG expression. Thirdly, as lncRNAs exerted their functions via acting as competing endogenous RNA (ceRNA) for microRNAs [34], further investigation should be performed to determine whether MIR503HG functioned as a ceRNA to regulate the cellular functions of trophoblasts. More importantly, MIR503HG is the host gene of miR-503 and MIR503HG could induce the miR-503 expression in lymphoma [35], while miR-503 has been well-documented for its regulatory role in cell invasion and migration [36]. As such, future investigations should focus on the effects of MIR503HG on the miR-503 expression to further reveal the underlying mechanisms of MIR503HG in PE pathogenesis.

## 5. Conclusions

Our results showed that MIR503HG inhibited proliferation, invasion, and migration of HTR-8/SVneo and JEG3 cells, and the inhibitory effect of MIR503HG was related to the downregulation of MMPs and suppression of the NF-*κ*B signaling pathway. The present study revealed the key role of MIR503HG in the invasive and migratory behaviors of trophoblasts, which may link to the pathogenesis of PE.

## Figures and Tables

**Table 1:** Clinical characteristics of pregnant women enrolled in this study.

Characteristics	Control (*n* = 40)	PE (*n* = 40)	*P* value
Prepregnancy BMI	21.6 ± 3.1	22.6 ± 2.5	0.1163
Maternal age (years old)	28.9 ± 3.5	29.1 ± 4.1	0.8151
Systolic blood pressure (mmHg)	114.8 ± 8.8	165.4 ± 5.3	*P* < 0.001
Diastolic blood pressure (mmHg)	78.9 ± 9.7	112.4 ± 11.4	*P* < 0.001
Proteinuria (g/24 h)	Nondetected	3.67 ± 1.5	*P* < 0.001
Current smoker	0	0	NA
Gestational age (weeks)	37.5 ± 1.4	36.9 ± 1.6	0.0782
Birth weight (g)	3178 ± 312.6	2401 ± 211.6	*P* < 0.001

BM: body mass index.

## Data Availability

The data used to support the findings of this study are available from the corresponding author upon request.
